# Cardiorespiratory fitness and body mass index on metabolic syndrome in middle-aged Japanese adults under national health guidance: a cross-sectional study

**DOI:** 10.1186/s12889-024-19544-0

**Published:** 2024-07-30

**Authors:** Xiangyu Zhai, Susumu S. Sawada, Sayaka Kurosawa, Sakura Koriyama, Serena A. Dimitroff, Shinji Sato, Yuko Oguma, Yoshio Nakata, Kazushi Maruo, Motohiko Miyachi, Yuko Gando, Koichiro Oka, Duck-chul Lee

**Affiliations:** 1 Graduate School of Sport Sciences, Waseda University, Saitama, Japan; 2 School of Nursing, Li Ka Shing Faculty of Medicine, The University of Hong Kong, Hong Kong Special Administrative Region, China; 3 Faculty of Sport Sciences, Waseda University, Saitama, Japan; 4 College of Sport and Wellness, Rikkyo University, Saitama, Japan; 5 Faculty of Medical Technology, Teikyo University, Tokyo, Japan; 6 Sports Medicine Research Center, Keio University, Kanagawa, Japan; 7 Institute of Health and Sport Sciences, University of Tsukuba, Ibaraki, Japan; 8 Department of Biostatistics, Institute of Medicine, University of Tsukuba, Ibaraki, Japan; 9 Faculty of Sport Science, Surugadai University, Saitama, Japan; 10 Department of Health and Human Development, University of Pittsburgh, Pittsburgh, PA USA

**Keywords:** Cardiorespiratory fitness, Body mass index, Metabolic syndrome, Epidemiology

## Abstract

**Objectives:**

Poor cardiorespiratory fitness (CRF) and high body mass index (BMI) increased the risk of developing metabolic Syndrome (MetS) mostly in Caucasians. However, the sex-specific combined association of CRF and BMI on MetS considering health-related behaviors has yet to be thoroughly examined in Japanese. This study aims to investigate the sex-specific independent and combined associations of CRF and BMI with MetS in middle-aged Japanese adults.

**Methods:**

421 participants were included in this cross-sectional study. CRF was estimated using a submaximal cycle ergometer. CRF and BMI were respectively divided into three categories according to tertile distribution. MetS was diagnosed based on five risk factors: waist circumference, triglycerides, high-density lipoprotein cholesterol, blood pressure, and fasting glucose. Multivariable logistic regression models were used to estimate independent and combined association of CRF and BMI with MetS.

**Results:**

Results showed that 154 (57.5%) and 70 (45.8%) of men and women had MetS, respectively. Compared to men with lower CRF or higher BMI, men with middle and higher CRF or middle and lower BMI were less likely to have MetS. Compared with ‘unfit and higher BMI’ group, ‘unfit and lower BMI’, ‘fit and higher BMI’, and ‘fit and lower BMI’ groups in men showed statistically significant decreased prevalences of MetS. However, no significant associations were found in women.

**Conclusions:**

This study found significant independent and combined associations of CRF and BMI with MetS only in men, but not in women. However, prospective studies are warranted to confirm sex-specific associations of CRF and BMI with MetS.

**Supplementary Information:**

The online version contains supplementary material available at 10.1186/s12889-024-19544-0.

## Introduction

Metabolic syndrome (MetS) is a clustering of hyperglycemia/insulin resistance, obesity, dyslipidemia, and high blood pressure that contributes to developing atherosclerotic cardiovascular disease (CVD), type 2 diabetes, and premature mortality [1]. The prevalence of MetS has been increasing worldwide [2–6], since the transition to modern lifestyles, including the increase in consumption of fast food and the decrease in physical activity [7, 8]. For example, the prevalence of MetS increased from 37.6% in 2011–12 to 41.8% in 2017–18, according to the data from the United States National Health and Nutrition Examination Survey [9].

Evidence shows that lifestyle modifications can effectively improve all components of MetS [10]. Healthy lifestyles can be reflected by higher cardiorespiratory fitness (CRF) and lower body mass index (BMI), which may attenuate MetS. It is well established that the combined association of poor fitness and fatness is least favorable to CVD mortality [11], but the relative and combined contributions of fitness and fatness to other health outcomes, including MetS are still controversial. Some studies suggested that obese individuals with a higher level of fitness do not have excess health problems since fitness can offset the adverse effects of obesity [12, 13]. However, others reported that while higher levels of fitness can offer certain benefits, they may not entirely mitigate the negative health impacts associated with obesity [14, 15].

Although the independent and combined associations of CRF and BMI with MetS have been reported primarily on Western populations [16–20], there are factors that limit the applicability of this knowledge to the Japanese population. A significant barrier is the variation in MetS prevalence across different geographic regions [21]. Despite having a lower BMI, Asians tend to store more visceral fat [22]; this “skinny-fat” Asian syndrome influenced by genetics and environment cannot be overlooked. Furthermore, the prevalence of MetS increases with advancing age and is common in middle-aged adults. Prevention is crucial before the development of CVD, diabetes, or premature mortality. In 2008, the Japanese Ministry of Health, Labour, and Welfare (MHLW) introduced a nationwide program to provide health guidance for individuals with CV risk factors but no meaningful clinical effects were found. Examining the relative importance of CRF or BMI and the potential additive benefits of both on MetS in the population under health guidance may contribute to providing suggestions for improving national health guidance and other public health policies.

Given that, in the general Japanese population aged over 40 years, the prevalence of MetS in men is thrice that in women [23], sex-specific associations are crucial and needed. Thus, the purpose of this study was to investigate the sex-specific independent and combined associations of CRF and BMI with MetS in middle-aged Japanese adults under national health guidance. It was hypothesized that: (1) lower CRF is associated with a higher prevalence of MetS, independent of BMI; (2) higher BMI is associated with a higher prevalence of MetS, independent of CRF; and (3) the combined association of CRF and BMI with prevalence of MetS would be stronger than the association of either CRF or BMI alone with MetS.

## Methods

### Participants

A cross-sectional study was conducted using observational data from the Japan Health Promotion Facility (JHPF) Study, supported by the Japanese MHLW. The JHPF Study, a 6-month randomized controlled trial to examine the physical and psychological effects of structured exercise training on middle-aged Japanese adults, was conducted at 18 Health Promotion Facilities located across Japan. All participants underwent a National Screening Program as outlined in Supplementary Fig. 1 established by MHLW [24], and only those who were eligible to be under intensive and moderate support from National Health Guidance were recruited in this study. After excluding the participants (*n* = 38) with missing information on alcohol drinking (*n* = 1), education levels (*n* = 4), sedentary time (*n* = 3), and physical activity (*n* = 9), blood pressure (*n* = 1), fasting glucose (*n* = 8), CRF (*n* = 13), and waist circumference (*n* = 1), a total of 421 participants aged 40 − 64 years old were included in this study. Written informed consents were obtained from all participants before the trial began. This study has been approved by the Ethics Committees of Waseda University (approval number: 2021 − 106) and conducted in accordance with the Helsinki Declaration and the Strengthening the Reporting of Observational Studies in Epidemiology (STROBE) reporting guideline.

### Measures

#### Cardiorespiratory fitness and body mass index

Participants underwent a submaximal exercise test on a cycle ergometer (V77i modified version; Seno Corp., Chiba, Japan) to assess CRF. The exercise test consisted of 5 or 6-minute progressively increasing exercise loads. Maximal oxygen uptake was estimated using Åstrand-Rhyming Nomogram and Åstrand’s Nomogram correction factors based on the exercise load and the heart rate [25, 26]. Participants were divided into three categories according to the distribution of tertile: lower (lower one-third), middle (middle one-third), and higher (higher one-third) CRF [27]. Body weight and height were measured on a standard scale (WB-150 S; TANITA Corp., Tokyo, Japan) with light clothing and a stadiometer (YS101-S; YOSHIDA Corp., Japan) without shoes. BMI was calculated using the formula: BMI = Weight (kg)/Height (m^2^). BMI was categorized as lower (lower one-third), middle (middle one-third), and higher (higher one-third) BMI according to tertile distribution.

#### Metabolic syndrome

Waist circumference was measured based on the middle of the bottom ribs and pelvic bones after a normal exhale using an inelastic tape. Fasting venous blood was collected to analyze biochemical variables such as glucose, triglycerides, and high-density lipoprotein cholesterol (HDL-C). Systolic and diastolic blood pressure were measured using automated sphygmomanometers in a sitting position. According to the International Diabetes Federation (IDF) and American Heart Association/National Heart, Lung, and Blood Institute (AHA/NHLBI) criteria, MetS was diagnosed as the presence of three of the following five risk factors: waist circumference ≥ 85 cm in men or ≥ 90 cm in women, elevated triglycerides ≥ 150 mg/dL, reduced HDL-C < 40 mg/dL in men or < 50 mg/dL in women, elevated systolic blood pressure ≥ 130 mm Hg and/or diastolic blood pressure ≥ 85 mm Hg, and elevated fasting glucose ≥ 100 mg/dL [28].

#### Covariates

A survey on participants’ sociodemographic characteristics, including age, sex, and education levels (junior high school graduates, high school graduates, junior, technical, or vocational college graduates, or college graduates or above), was conducted in this study. A self-reported questionnaire related to lifestyle, including sleep duration, smoking status (never, previous, or current), alcohol drinking frequency (never, sometimes, or every day), physical activity (≥ 600 MET-min/week or not), and sedentary behavior (> 7 h/day or not), was completed by participants.

### Statistical analysis

Descriptive characteristics were presented in this study, separated by sex. Continuous or categorical variables were expressed using means and standard deviations (SD) or numbers and percentages. ANOVA was used to examine group differences in each continuous variable across tertile of CRF and BMI in men and women, respectively. The chi-square test was used to examine group differences in each categorical variable across tertile of CRF and BMI in men and women, respectively. Four combined groups of CRF and BMI were created, including “unfit and higher BMI”, “unfit and lower BMI”, “fit and higher BMI”, and “fit and lower BMI”. These groups were defined based on the lower one-third of CRF and higher one-third of BMI for “unfit and higher BMI”, the lower one-third of CRF and lower two-third of BMI” for “unfit and lower BMI”, the higher two-third of CRF and higher one-third of BMI for “fit and higher BMI”, and the higher two-third of CRF and lower two-third BMI for “fit and lower BMI”. Multivariable logistic regression models were used to estimate the odds ratios (ORs) and 95% confidence intervals (95% CIs) of MetS across CRF (3 categories), BMI (3 categories), or the combination of CRF and BMI (4 groups). The models were adjusted for age, smoking status, alcohol drinking frequency, sleeping duration, physical activity, sedentary behavior, and education levels. All statistical analyses were conducted using R (4.1.1 version), with the acceptable threshold of statistical significance being specified as 0.05 (two-tailed).

## Results

Participants’ characteristics are presented according to tertiles of CRF and BMI separated by sex in Table 1. A total of 421 participants were included in this study, with 268 men (age: 50.5 ± 6.7) and 153 women (age: 50.0 ± 5.8). The mean BMI of men was 27.1 ± 3.1 kg/m^2^, which is lower than that of women (28.7 ± 3.4). The mean peak oxygen uptake of men was 28.1 ± 8.0 mL/kg/min, which is higher than that of women (25.6 ± 5.7). Among them, 154 (57.5%) men and 70 (45.8%) women were diagnosed with MetS. All participants in higher CRF group showed higher BMI, but only women in higher CRF were more likely to be more physically active and have less sitting time. Men with higher BMI were more likely to be younger and experience shorter durations of sleep. Women with higher BMI were more likely to have lower CRF.

**Table 1:** Characteristics of the study participants according to tertiles of CRF and BMI.

			Tertile of CRF					Tertile of BMI			
		Total	Lower	Middle	Upper	*p* value		Lower	Middle	Higher	*p* value
Men	Age (years)	50.5 ± 6.7	51.2 ± 6.6	50.4 ± 7.0	49.9 ± 6.6	0.468		52.5 ± 6.6	49.8 ± 6.6	49.2 ± 6.5	**0.002**
*n* = 268	Height (cm)	172.0 ± 5.7	172.2 ± 5.9	171.5 ± 5.7	172.2 ± 5.6	0.680		173.7 ± 5.7	171.4 ± 5.6	170.8 ± 5.5	**0.002**
	Weight (kg)	80.3 ± 10.2	82.8 ± 13.0	80.3 ± 8.2	77.8 ± 7.9	**0.004**		73.3 ± 5.1	78.1 ± 5.7	89.6 ± 10.7	**< 0.001**
	BMI (kg/m^2^)	27.1 ± 3.1	27.9 ± 3.6	27.3 ± 2.8	26.2 ± 2.5	**0.001**		24.3 ± 1.0	26.6 ± 0.7	30.6 ± 2.4	**< 0.001**
	Peak oxygen uptake (mL/kg/min)	28.1 ± 8.0	19.6 ± 4.8	28.2 ± 1.6	36.4 ± 5.1	**< 0.001**		28.9 ± 6.7	28.6 ± 8.8	26.8 ± 8.4	0.190
	Sleep duration (min)	380.3 ± 62.9	381.2 ± 70.5	375.4 ± 65.0	384.2 ± 52.3	0.637		392.2 ± 66.5	379.4 ± 57.5	369.1 ± 62.9	**0.048**
	Meeting PA recommendations (≥ 600 MET-min/week)	128 (47.8%)	40 (44.9%)	39 (43.8%)	49 (54.4%)	0.294		36 (40.0%)	46 (51.7%)	46 (51.7%)	0.195
	Sedentary time (> 7 h/day)	143 (53.4%)	41 (46.1%)	50 (56.2%)	52 (57.8%)	0.236		47 (52.2%)	49 (55.1%)	47 (52.8%)	0.923
	Smoking status					0.709					0.238
	Non-smoker	85 (31.7%)	26 (29.2%)	31 (34.8%)	28 (31.1%)			21 (23.3%)	31 (34.8%)	33 (37.1%)	
	Previous smoker	108 (40.3%)	34 (38.2%)	34 (38.2%)	40 (44.4%)			43 (47.8%)	35 (39.3%)	30 (33.7%)	
	Current smoker	75 (28.0%)	29 (32.6%)	24 (27.0%)	22 (24.4%)			26 (28.9%)	23 (25.8%)	26 (29.2%)	
	Alcohol drinking frequency					**0.023**					0.075
	Never	85 (31.7%)	40 (44.9%)	24 (27.0%)	21 (23.3%)			29 (32.2%)	23 (25.8%)	33 (37.1%)	
	Sometimes	86 (32.1%)	23 (25.8%)	32 (36.0%)	31 (34.4%)			21 (23.3%)	34 (38.2%)	31 (34.8%)	
	Every day	97 (36.2%)	26 (29.2%)	33 (37.1%)	38 (42.2%)			40 (44.4%)	32 (36.0%)	25 (28.1%)	
	Education levels					0.645					0.251
	College graduates or over	109 (40.7%)	34 (38.2%)	34 (38.2%)	41 (45.6%)			35 (38.9%)	43 (48.3%)	31 (34.8%)	
	Junior, technical, or vocational college graduates	38 (14.2%)	13 (14.6%)	16 (18.0%)	9 (10.0%)			10 (11.1%)	11 (12.4%)	17 (19.1%)	
	High school graduates	111 (41.4%)	37 (41.6%)	37 (41.6%)	37 (41.1%)			42 (46.7%)	30 (33.7%)	39 (43.8%)	
	Junior high school graduates	10 (3.7%)	5 (5.6%)	2 (2.2%)	3 (3.3%)			3 (3.3%)	5 (5.6%)	2 (2.2%)	
	MetS	154 (57.5%)	62 (69.7%)	52 (58.4%)	40 (44.4%)	**0.003**		43 (47.8%)	51 (57.3%)	60 (67.4%)	**0.029**
	Waist circumference (cm)	95.8 ± 7.5	98.1 ± 8.7	95.9 ± 6.8	93.5 ± 6.1	**< 0.001**		91.4 ± 3.7	93.6 ± 4.6	102.6 ± 8.0	**< 0.001**
	Triglycerides (mg/dL)	169.3 ± 112.9	183.9 ± 128.6	176.9 ± 110.8	147.4 ± 94.6	0.071		145.9 ± 77.3	185.9 ± 135.5	176.4 ± 115.5	**0.046**
	HDL cholesterol (mg/dL)	53.5 ± 12.6	51.3 ± 12.1	52.1 ± 11.5	57.2 ± 13.5	**0.003**		56.2 ± 13.5	53.3 ± 13.2	50.9 ± 10.5	**0.020**
	Systolic blood pressure (mmHg)	128.0 ± 14.3	129.1 ± 14.9	128.6 ± 14.5	126.2 ± 13.5	0.352		125.7 ± 14.4	127.2 ± 13.7	131.1 ± 14.4	**0.031**
	Diastolic blood pressure (mmHg)	82.7 ± 11.1	84.3 ± 11.8	83.0 ± 11.1	80.9 ± 10.3	0.118		82.0 ± 11.8	81.7 ± 10.2	84.6 ± 11.3	0.163
	Fasting glucose (mg/dL)	104.5 ± 17.2	105.0 ± 13.6	105.1 ± 13.5	103.4 ± 22.9	0.759		102.6 ± 13.8	104.0 ± 13.9	107.0 ± 22.5	0.215
Women	Age (years)	50.0 ± 5.8	50.8 ± 6.0	48.8 ± 4.9	50.3 ± 6.3	0.192		51.3 ± 5.6	49.4 ± 6.2	49.2 ± 5.5	0.132
*n* = 153	Height (cm)	158.8 ± 5.1	158.3 ± 5.5	159.5 ± 4.5	158.7 ± 5.2	0.442		159.9 ± 4.5	158.2 ± 5.6	158.4 ± 5.1	0.171
	Weight (kg)	72.5 ± 9.4	75.5 ± 10.0	72.3 ± 7.8	69.7 ± 9.6	**0.007**		65.2 ± 4.1	70.7 ± 5.8	81.5 ± 8.9	**< 0.001**
	BMI (kg/m^2^)	28.7 ± 3.4	30.2 ± 4.0	28.4 ± 2.7	27.6 ± 2.9	**< 0.001**		25.5 ± 1.0	28.2 ± 0.8	32.5 ± 3.0	**< 0.001**
	Peak oxygen uptake (mL/kg/min)	25.6 ± 5.7	19.2 ± 3.2	25.9 ± 1.4	31.7 ± 2.7	**< 0.001**		27.7 ± 5.5	25.7 ± 6.2	23.5 ± 4.7	**0.001**
	Sleep duration (min)	365.7 ± 59.6	368.6 ± 63.5	370.2 ± 52.4	358.6 ± 62.6	0.565		364.7 ± 55.1	371.2 ± 64.6	361.3 ± 59.5	0.698
	Meeting PA recommendations (≥ 600 MET-min/week)	46 (30.1%)	16 (31.4%)	9 (18.0%)	21 (40.4%)	**0.046**		12 (23.5%)	15 (29.4%)	19 (37.3%)	0.317
	Sedentary time (> 7 h/day)	76 (49.7%)	34 (66.7%)	25 (50.0%)	17 (32.7%)	**0.003**		25 (49.0%)	20 (39.2%)	31 (60.8%)	0.093
	Smoking					0.726					0.714
	Non-smoker	111 (72.5%)	39 (76.5%)	35 (70.0%)	37 (71.2%)			36 (70.6%)	39 (76.5%)	36 (70.6%)	
	Previous smoker	27 (17.6%)	8 (15.7%)	8 (16.0%)	11 (21.2%)			10 (19.6%)	6 (11.8%)	11 (21.6%)	
	Current smoker	15 (9.8%)	4 (7.8%)	7 (14.0%)	4 (7.7%)			5 (9.8%)	6 (11.8%)	4 (7.8%)	
	Alcohol drinking frequency					0.690					0.931
	Never	80 (52.3%)	29 (56.9%)	27 (54.0%)	24 (46.2%)			26 (51.0%)	25 (49.0%)	29 (56.9%)	
	Sometimes	46 (30.1%)	12 (23.5%)	15 (30.0%)	19 (36.5%)			15 (29.4%)	17 (33.3%)	14 (27.5%)	
	Every day	27 (17.6%)	10 (19.6%)	8 (16.0%)	9 (17.3%)			10 (19.6%)	9 (17.6%)	8 (15.7%)	
	Education levels					0.165					0.168
	College graduates or over	29 (19.0%)	9 (17.6%)	8 (16.0%)	12 (23.1%)			6 (11.8%)	10 (19.6%)	13 (25.5%)	
	Junior, technical, or vocational college graduates	59 (38.6%)	16 (31.4%)	17 (34.0%)	26 (50.0%)			19 (37.3%)	23 (45.1%)	17 (33.3%)	
	High school graduates	60 (39.2%)	25 (49.0%)	23 (46.0%)	12 (23.1%)			22 (43.1%)	18 (35.3%)	20 (39.2%)	
	Junior high school graduates	5 (3.3%)	1 (2.0%)	2 (4.0%)	2 (3.8%)			4 (7.8%)	0 (0%)	1 (2.0%)	
	MetS	70 (45.8%)	27 (52.9%)	19 (38.0%)	24 (46.2%)	0.320		19 (37.3%)	24 (47.1%)	27 (52.9%)	0.275
	Waist circumference (cm)	97.2 ± 7.7	100.5 ± 8.1	96.3 ± 6.4	94.9 ± 7.5	**< 0.001**		92.2 ± 4.3	95.5 ± 5.7	103.9 ± 7.5	**< 0.001**
	Triglycerides (mg/dL)	120.6 ± 61.2	118.1 ± 56.3	126 ± 72.5	117.8 ± 54.4	0.751		120.7 ± 70.5	121.6 ± 47.3	119.4 ± 64.7	0.983
	HDL cholesterol (mg/dL)	62.3 ± 13.9	61.7 ± 14.2	60.6 ± 13.2	64.4 ± 14.2	0.372		64.0 ± 12.9	61.3 ± 16.1	61.5 ± 12.5	0.559
	Systolic blood pressure (mmHg)	127.9 ± 15.2	129.5 ± 16.5	125.7 ± 15.3	128.3 ± 13.7	0.438		127.3 ± 15.1	124.1 ± 15.5	132.3 ± 14.1	**0.023**
	Diastolic blood pressure (mmHg)	79.5 ± 10.7	80.9 ± 10.9	77.7 ± 11.7	79.9 ± 9.5	0.311		80.0 ± 12.2	76.8 ± 9.7	81.8 ± 9.7	0.059
	Fasting glucose (mg/dL)	102.9 ± 10.5	102.6 ± 11.2	103.6 ± 11.5	102.6 ± 8.8	0.864		101.4 ± 8.3	104.2 ± 10.4	103.2 ± 12.3	0.395

Data are expressed as means ± standard deviation or number and percentages of participants

CRF, cardiorespiratory fitness; BMI, body mass index; HDL, high-density lipoprotein; PA, physical activity; MetS, metabolic syndrome

The sex-specific cutpoints based on the distribution of tertiles for lower, middle, and higher CRF measured by cycle ergometer were < 25.1, 25.1–30.7, and ≥ 30.8 for men and < 23.6, 23.6 − 28.4, and ≥ 28.5 for women, respectively

The sex-specific cutpoints based on the distribution of tertiles for lower, middle, and higher BMI were ≤ 25.6, 25.7–28.0, and > 28.0 for men and ≤ 26.7, 26.7–29.7, and ≥ 29.7 for women, respectively

Additionally, men with lower CRF or higher BMI had a higher prevalence of MetS than those with higher CRF or lower BMI. The mean and SDs of MetS indicators across the three incremental CRF levels are presented. The waist circumference of participants with higher CRF was significantly lower than those with middle and lower CRF in both men and women. The HDL cholesterol of participants with higher CRF were significantly greater than those with middle and lower CRF in men. Across the three incremental BMI levels, the waist circumference and systolic blood pressure of participants with higher BMI were significantly higher than those with lower BMI in both men and women. The triglycerides and HDL cholesterol of men with lower BMI were significantly better than those with higher BMI.

The results of logistic regression that examined the independent relationship between CRF and BMI with MetS are shown in Table 2. Compared to men with lower CRF, men with middle (OR = 0.52, 95% CI = 0.26–1.00) and higher (OR = 0.33, 95% CI = 0.17–0.65) CRF had higher prevalences of MetS after adjusting for potential covariates including BMI. Compared to men with higher BMI, ORs (95% CIs) were 0.63 (0.32–1.21) and 0.42 (0.21–0.82) in men with middle and lower BMI, respectively, after adjusting for potential covariates including CRF. In women, however, neither CRF nor BMI was associated with MetS.

**Table 2:** Odds ratios for MetS according to independent tertiles of CRF and BMI separated by sex

		*n*	cases (%)	Model 1 OR (95% CI)	Model 2 OR (95% CI)	Model 3 OR (95% CI)
Men	CRF					
	Lower	89	62 (69.7)	1.00 (reference)	1.00 (reference)	1.00 (reference)
	Middle	89	52 (58.4)	0.62 (0.33 − 1.15)	0.49 (0.25–0.95)	0.52 (0.26 − 1.00)
	Higher	90	40 (44.4)	**0.35 (0.19 − 0.65)**	**0.28 (0.14 − 0.55)**	**0.33 (0.17 − 0.65)**
	BMI					
	Higher	89	60 (67.4)	1.00 (reference)	1.00 (reference)	1.00 (reference)
	Middle	89	51 (57.3)	0.64 (0.34 − 1.17)	0.58 (0.31 − 1.11)	0.63 (0.32 − 1.21)
	Lower	90	43 (47.8)	**0.40 (0.21 − 0.75)**	**0.37 (0.19 − 0.73)**	**0.42 (0.21 − 0.82)**
Women	CRF					
	Lower	51	24 (46.2)	1.00 (reference)	1.00 (reference)	1.00 (reference)
	Middle	50	19 (38.0)	0.61 (0.27 − 1.38)	0.58 (0.24 − 1.40)	0.66 (0.27 − 1.63)
	Higher	52	27 (52.9)	0.78 (0.35 − 1.71)	0.73 (0.30 − 1.78)	0.90 (0.34 − 2.35)
	BMI					
	Higher	51	27 (52.9)	1.00 (reference)	1.00 (reference)	1.00 (reference)
	Middle	51	24 (47.1)	0.76 (0.34 − 1.70)	0.77 (0.33 − 1.84)	0.79 (0.33 − 1.90)
	Lower	51	19 (37.3)	0.43 (0.19–0.97)	0.43 (0.18 − 1.04)	0.45 (0.18 − 1.14)

CRF, cardiorespiratory fitness; BMI, body mass index; OR, odds ratio; CI, confidence interval; MetS, metabolic syndrome

Model 1: adjusted for age

Model 2: model 1 and further adjusted for smoking status, alcohol drinking frequency, sleep duration, physical activity, sedentary behavior, and education levels

Model 3: model 2 and further adjusted for BMI for CRF or CRF for BMI

**Fig. 1:**
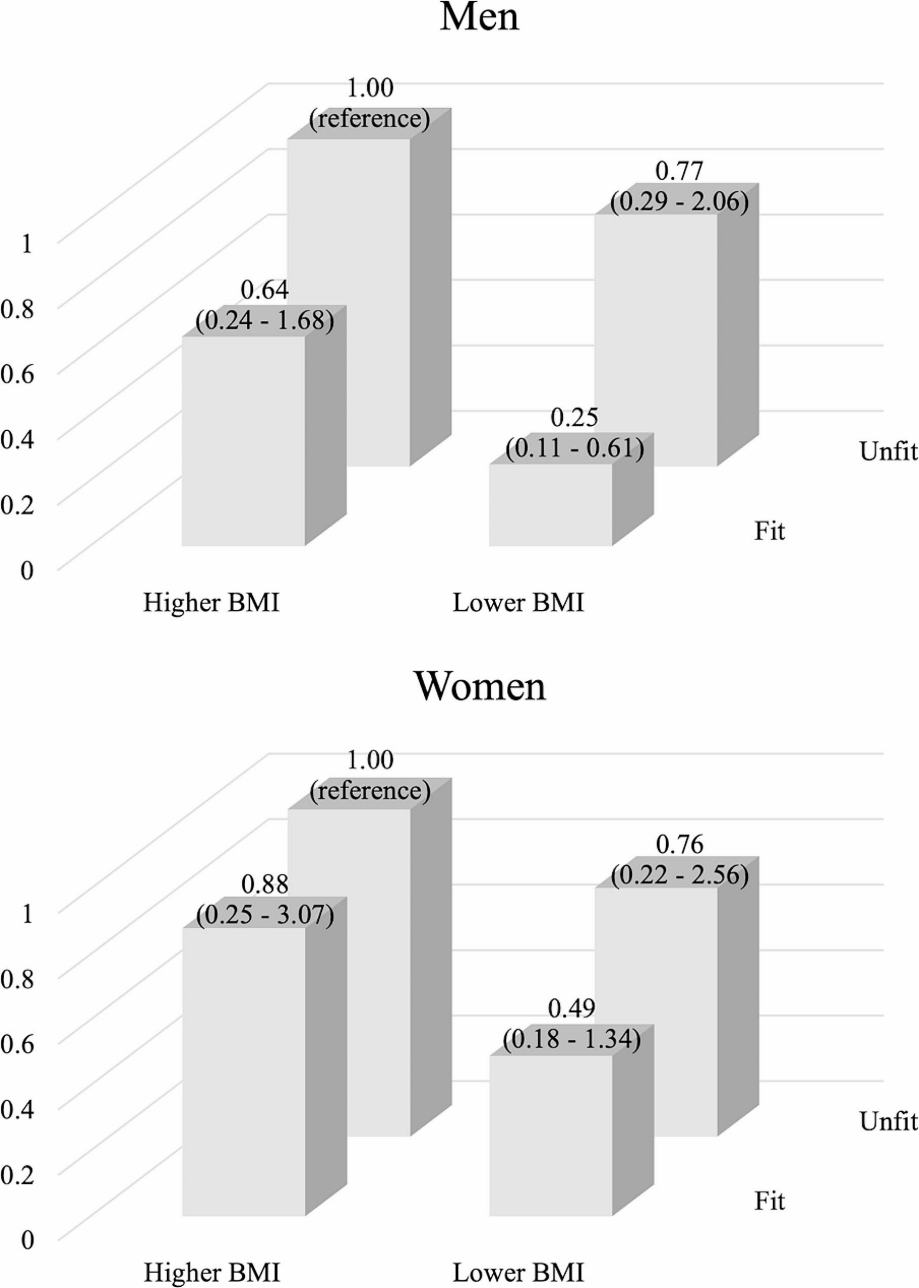
Odds ratios for metabolic syndrome according to combined tertiles of cardiorespiratory fitness and body mass index separated by sex. The model was adjusted for age, smoking status, alcohol drinking frequency, sleep duration, physical activity, sedentary behavior, and education levels

Figure 1 shows the ORs (95% CIs) of MetS according to the combinations of two CRF categories (fit and unfit) and two BMI categories (higher and lower BMI). In men, compared with ‘unfit and higher BMI’ group, the prevalence of MetS decreased in ‘unfit and lower BMI’, ‘fit and higher BMI’, and ‘fit and lower BMI’ groups, after adjusting for potential covariates. The lowest prevalence of MetS was observed in men with lower BMI who were fit (OR = 0.25, 95% CI = 0.11–0.61). When directly comparing ‘fit and higher BMI’ to men with ‘unfit and lower BMI’, there was no significant difference in the prevalence of MetS in men (*P* = 0.365). However, no significant combined association was found in women.

## Discussion

The primary finding of this study was that higher CRF and lower BMI are both independently associated with a lower prevalence of MetS in middle-aged Japanese men only, but not in women. Additionally, a combined analysis revealed that there is an additive association of CRF and BMI with the lowest prevalence observed in men with ‘higher CRF and lower BMI.’ However, the association of CRF and BMI appears to be similar when directly comparing the “fit and higher BMI” group with the “unfit and lower BMI” group in men.

The Aerobics Center Longitudinal Study (ACLS) and Nord-Trøndelag Health Study (the HUNT Study) found that low CRF and high fatness are strong independent predictors of incident MetS in both men and women [20, 29–32]. The inconsistency between the ACLS or HUNT study and the current study may be due to different races. The population in ACLS consisted primarily of White adults, while this study is in Japanese (Asian). Another speculation of sex-specific is participants in this study consisted of middle-aged adults, which means some women were in the period of menopausal transition. This situation may lead to variation in menopausal status among women participants, with some already experiencing menopause while others had not yet entered this stage. Based on previous evidence, women with menopause had a significantly higher relative risk of MetS [33, 34]; we did not observe associations of CRF and BMI with MetS in women, possibly due to the absence of menopausal status data.

In addition, potential variations in health status between Japanese men and women should be considered. The percentage of Japanese men meeting the criteria for receiving health guidance was 17.3%, nearly double the 9.1% of Japanese women [35], suggesting that Japanese women may have a better health status than men. While all participants in this study were under health guidance provided by physicians, public health nurses, or dietitians, it is possible that women exhibited greater adherence to health guidance compared to men. For example, compared to men, women had healthier lifestyles than men, as evidenced by their lower rates of smoking status, alcohol drinking frequency, and sedentary behavior, with the exception of physical activity. Our findings, to some extent, can contribute to sex-specific health promotion policy-making. However, prospective studies are clearly warranted to confirm sex-specific associations of CRF and BMI with developing MetS.

Although previous studies reported the relationship between low CRF and MetS in Japanese workers, the CRF was evaluated using step tests or self-reported physical activity rather than objectively measured fitness [36, 37]. One concern regarding self-reported physical activity measurement is that people seem to be prone to overestimate their physical activity level [38], leading to inaccurate CRF estimation. Another study in Japan examined the association between CRF and MetS using a cycle ergometer, but only men aged 20–64 years were included in this study, and all of them were recruited in one city (Ibaraki) [39]. To the best of our knowledge, our study is the first to recruit participants from across Japan to examine the association of CRF and BMI with MetS in both middle-aged women and men. Given that the high prevalence of risk factors for cardiovascular disease, such as obesity, physical inactivity, and poor diet, has been observed among young individuals living in developed countries in the past two decades [40], prospective studies on middle-aged adults are clearly warranted to confirm sex-specific associations of CRF and BMI with developing MetS since the adverse hazards of cardiovascular risks may come before entering old age with modern lifestyle transition.

CRF and BMI are modifiable factors, and there is substantial evidence supporting that engaging in regular exercise can effectively reduce the risk of MetS by enhancing CRF and reducing BMI. A study including men and women with MetS indicated that their VO_2peak_ increased while body weight decreased after a 16-week exercise intervention, and 37% and 46% of the patients in moderate continuous-training group and aerobic interval-training group no longer met the criteria for a MetS diagnosis [41–44]. It is worth noting that Japan is one of the countries with the lowest obesity prevalence (< 5%) [45], which may lead individuals to overestimate their health status based on their normal weight, potentially causing an oversight of their fitness status. Our findings underscore the significance of not just higher BMI, but also lower CRF associated with a higher prevalence of MetS in men, which suggests that interventions such as regular exercise and physical activity should be promoted to increase CRF and reduce BMI to lower the prevention of MetS, especially in middle-aged Japanese men.

The main strength of this study is that the participants were recruited from across Japan, which enhances the generalizability and representation of middle-aged Japanese adults under health guidance. We also performed all analyses in men and women separately to see sex-specific differences in the associations of CRF and BMI with MetS. In addition, several health-related behaviors, including sleep duration, tobacco and alcohol use, physical activity, and sedentary behavior, were included as covariates since they could have been associated with CRF, BMI, and MetS. The CRF in this study was objectively measured from heart rate during submaximal exercise using the Åstrand-Rhyming Nomogram and Åstrand’s Nomogram correction factors [25, 26]. However, the method used, which relies on the estimation of maximum oxygen uptake, has been validated as highly correlated with direct CRF measurements [46, 47]. The major limitation is that the causal inference cannot be made due to the cross-sectional study design. Additionally, data on body fat composition, which is more accurate to reflect fatness than BMI, were not collected. The lack of information on menopausal status in women may have biased the results through the potential effects on MetS. The applicability of this study may be specific to persons under National Health Guidance only.

## Conclusion

We found that lower CRF and higher BMI, even after controlling for each other, are significantly associated with the prevalence of MetS in middle-aged Japanese men, but not women. In addition, the relative contribution of high CRF and low BMI appears to be similar to the prevalence of MetS in men. Therefore, it is important to promote targeted and tailored intervention programs to lower BMI and promote CRF in order to lower the prevalence of MetS in men. However, prospective studies are clearly warranted to confirm sex-specific associations of CRF and BMI with developing MetS.

### Electronic supplementary material

Below is the link to the electronic supplementary material.

Supplementary Material 1

## Data Availability

The datasets used and/or analyzed during the current study are available from the corresponding author upon reasonable request.

## Abbreviations

CRF: Cardiorespiratory fitness
BMI: Body mass index
MetS: Metabolic syndrome
CVD: cardiovascular disease
JHPF: Japan Health Promotion Facility
MHLW: Ministry of Health, Labour and Welfare
STROBE: Strengthening the Reporting of Observational Studies in Epidemiology
HDL-C: High-density lipoprotein cholesterol
IDF: International Diabetes Federation
AHA: American Heart Association
NHLBI: National Heart, Lung, and Blood Institute
SD: Standard deviations
OR: Odds ratio
CI: Confidence interval
ACLS: Aerobics Center Longitudinal Study
HUNT: Nord-Trøndelag Health Study
