# Supplementary material for: Cardiorespiratory fitness and body mass index on metabolic syndrome in middle-aged Japanese adults under national health guidance: a cross-sectional study
Source: BMC Public Health. 2024 Jul 30;24:2050. doi: 10.1186/s12889-024-19544-0 (PMC11290158; doi:10.1186/s12889-024-19544-0)
Supplement: Supplementary file 1 — Supplementary Material 1 [file 12889_2024_19544_MOESM1_ESM.docx]

Supplement


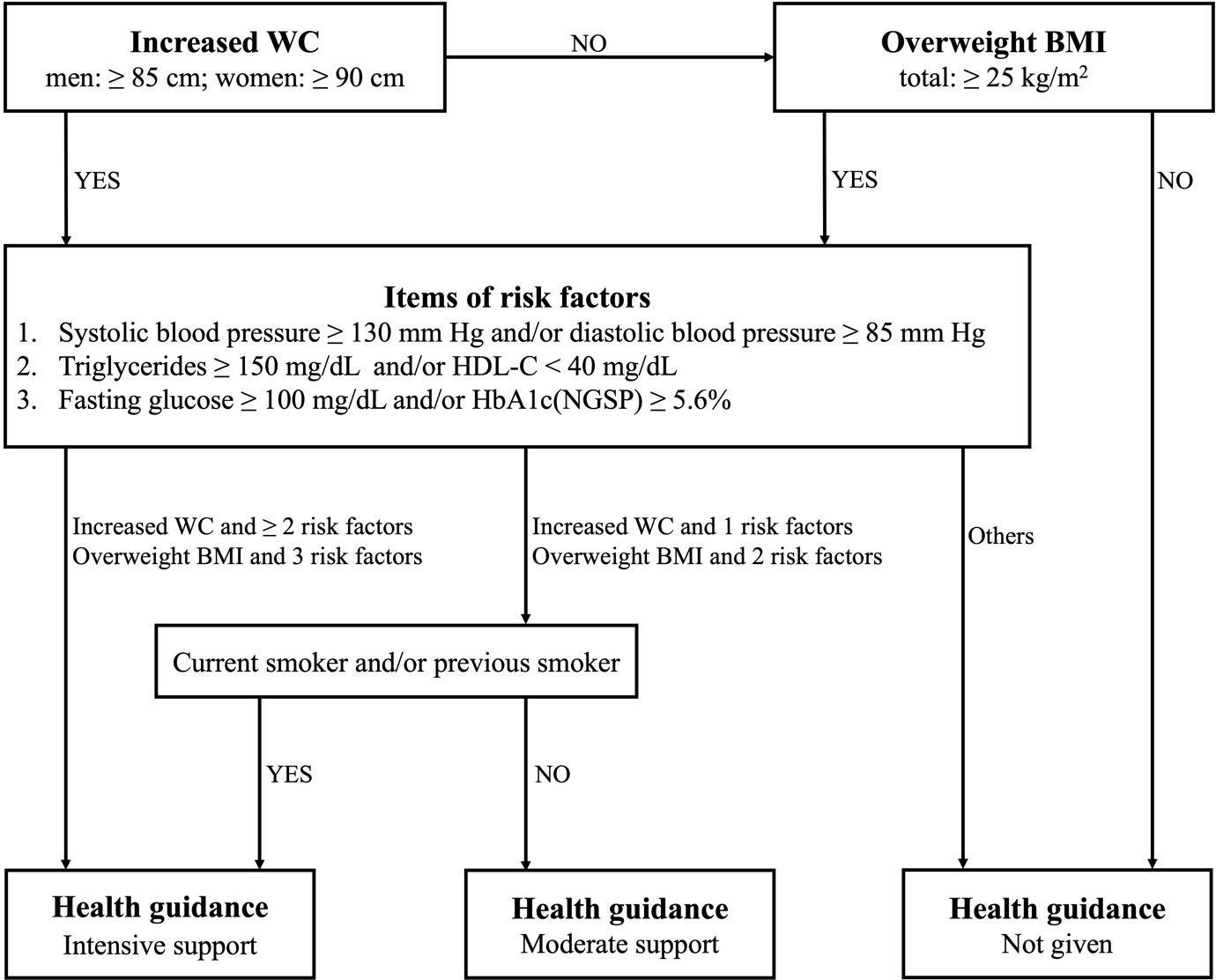


Figure 1 Flow of National Screening Program and Health Guidance.

Individuals receiving medications for hypertension, diabetes, and dyslipidemia were excluded from National Health Guidance.

WC, waist circumference; BMI, body mass index
